# Do risk-prone behaviours compromise reproduction and increase vulnerability of fish aggregations exposed to fishing?

**DOI:** 10.1098/rsbl.2024.0292

**Published:** 2024-08-07

**Authors:** Rucha Karkarey, L Boström Einarsson, Nicholas A. J. Graham, Ibrahim Mukrikkakudi, Mohammed Nowshad Bilutheth, Abdul Riyas Chekkillam, Idrees Babu KK, Sally A. Keith

**Affiliations:** ^1^ Lancaster Environment Centre, Lancaster University, Lancaster LA1 4YQ, UK; ^2^ Mukrikkakudi House, Kadmat, Lakshadweep, India; ^3^ Research and Environmental Education Foundation (REEF), Kavaratti, Lakshadweep, India; ^4^ Department of Science and Technology, Kavaratti, Lakshadweep, India

**Keywords:** anti-predator responses, mating aggregations, human disturbance, behavioural trade-offs, risk-prone behaviours, risk-averse behaviours

## Abstract

Human disturbances can prompt natural anti-predator behaviours in animals, affecting how energy is traded off between immediate survival and reproduction. In our study of male squaretail groupers (*Plectropomus areolatus*) in India’s Lakshadweep archipelago, we investigated the impact of fishing pressure on anti-predatory responses and reproductive behaviours by comparing a fished and unfished spawning aggregation site and tracking responses over time at the fished site. Using observational sampling and predator exposure experiments, we analysed fear responses (flight initiation distance, return time), as well as time spent in vigilance, courtship and territorial defence. Unpaired males at fished sites were twice as likely to flee from simulated predators and took longer to return to mating territories. In contrast, paired males at both sites took greater risks during courtship, fleeing later than unpaired males, but returned earlier at the unfished site compared with the fished site. Our findings suggest that high fishing pressure reduces reproductive opportunities by increasing vigilance and compromising territorial defence, potentially affecting mate selection cues. Altered behavioural trade-offs may mitigate short-term capture risk but endanger long-term population survival through altered reproductive investment. Human extractive practices targeting animal reproductive aggregations can have disruptive effects beyond direct removal, influencing animal behaviours crucial for population survival.

## 1. Introduction

‘Fear’ or predation risk influences animal behaviour, affecting mating, foraging and social interactions [1,2]. Human disturbances can prompt natural predator avoidance behaviours in animals [3]. Animals often balance multiple behaviours in response to predation risk, which varies within and between species and with ecological context [4]. Life-history theory suggests that breeding individuals adjust to perceived predation risk by reallocating reproductive investment to other behaviours [5,6]. In species that exhibit seasonal mating aggregations, which often represent exclusive breeding periods for certain populations, human disturbance can result in all reproductive individuals simultaneously being exposed to similar perceived risks. Therefore, it is crucial to unravel the behavioural trade-offs that aggregating animals make in response to predation risk. These behavioural trade-offs can have profound consequences for long-term population dynamics [1,7] particularly in species already threatened by human activities like hunting and habitat loss.

Fear responses can be reactive or proactive. Immediate threats can trigger acute fear, leading to reactive anti-predator responses such as flight [4], sheltering [8] and nest and territory abandonment [9]. Even in the absence of direct or immediate threats, chronic fear, prompting learned or evolved responses to cues, can influence proactive behaviours such as increased risk assessment or vigilance [10]. Predation risk is hypothesized to increase the immediate costs of engaging in reproductive behaviours such as sexual signalling, mate searching and male–male aggression, because individuals exhibiting these behaviours may be more conspicuous to predators, leading to greater investment in vigilance behaviours [11,12]. The optimal escape theory predicts that prey counterbalance risks and costs to decide the least energetically costly moment to escape from predators [13]. While escaping early can ensure survival, escaping too early may lead to significant costs in the form of lost mating opportunities. Fear effects have been largely studied in social and foraging contexts but there is a significant knowledge gap in understanding the ecology of fear in reproductive contexts. Bridging this gap is essential as reproduction has an immediate consequence for population regeneration and dynamics.

Many commercially important coral reef fish of the grouper–snapper complex form large, spatially and temporally specific spawning aggregations [14]. Historical observations in Palau in 1981 noted a ‘spawning stupor’ at grouper and snapper aggregation sites, where individuals, engrossed in spawning, exhibited minimal reaction to natural predators [15]. Exploiting this behaviour, fisheries have targeted spawning aggregations, contributing to their global decline [16]. On the one hand, fear effects such as increased flightiness and decreased aggression have been documented in fish exposed to fishing outside of the reproductive context [16,17]. On the other hand, controlled laboratory experimental studies on freshwater species of fish like guppies and sticklebacks have linked fear with variation in mate choice [18] and sexual signalling [19] but little is known about fear effects in natural mating systems like spawning aggregations. Individuals in spawning aggregations potentially contend with the dual challenge of compromised survival and reproductive success amid human-induced pressures, making these ideal and urgent systems to study fear effects.

In this study, we test the extent to which anti-predator responses disrupt the reproductive behaviour of squaretail groupers (*Plectropomus areolatus*) in spawning aggregations exposed to different levels of fishing in the lightly fished Lakshadweep archipelago, off the southwest coast of India (electronic supplementary material, figure S1). Spawning aggregations are rare in both space and time and are highly fished where known. As a result, we have limited knowledge at present of their ecological dynamics in the absence of fishing, despite their critical importance for population survival. Here, we present a rare example of behavioural data from two spawning aggregations—one at a fished island and the other at a relatively ‘unfished’ island within the same archipelago—to disentangle the effects of fishing pressure on reproductive behaviour. To determine whether fishing pressure, rather than other site-level differences, is the main driver of behavioural changes, we analyse how behaviours have shifted over time at the fished site and compare this to variations among sites with different fishing exposures. Using observations and experimental simulation of predation risk, we tested whether reactive (flight initiation distance (FID), territory return duration) and proactive behavioural responses (i.e. differences in time–activity budgets) differed among and within sites. We hypothesized that individuals at the fished site (i.e. with increasing exposure to fishing) would be more likely to flee early [16] and show heightened vigilance, potentially at the cost of courtship and interspecific aggression [17]. We expected individual courtship status (paired, unpaired) to impact individual reactive and proactive fear irrespective of fishing.

**Figure 1.**
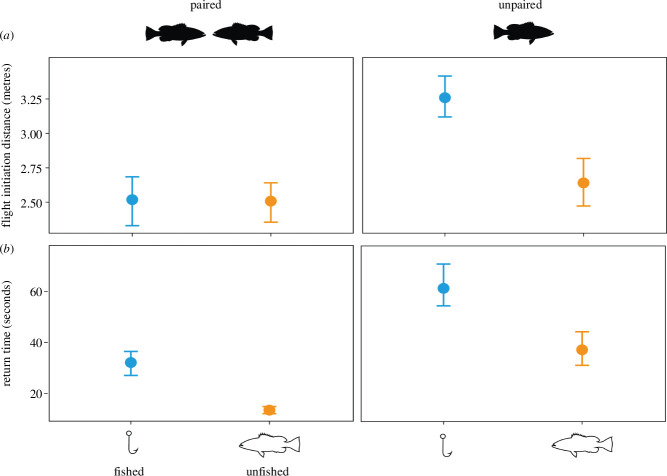
Reactive response: (*a*) FID (metres) comparison between paired and unpaired males (*n* = 72) at fished (blue, Bitra in 2023–2024) and unfished (yellow, Site 2 in 2023–2024) sites. (*b*) Return time (s) for males to reclaim territories after simulated disturbance. Points represent means, whiskers display 95% bootstrapped confidence intervals.

**Figure 2.**
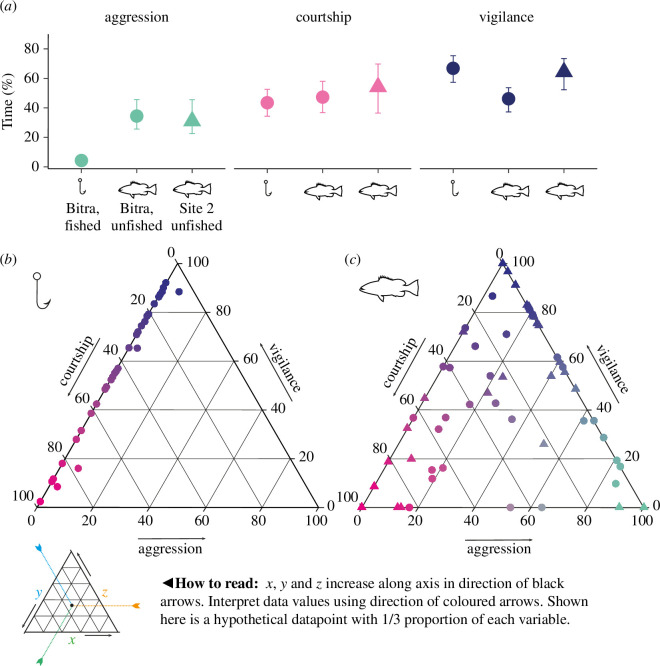
Proactive response: (*a*) male time–activity budgets at Bitra (circles) in fished (2023–2024) and unfished (2013–2014) states, and at Site 2 (triangles). Plots display means with 95% bootstrapped confidence intervals. Ternary plots illustrating behaviour trade-offs at (*b*) fished (Bitra in 2023–2024) and (*c*) unfished (Bitra in 2013/2014 and Site 2 in 2023–2024) sites. Each point represents an individual male (*n* = 108), with colour indicating dominant activity (green = aggression, pink = courtship, purple = vigilance).

## 2. Methods

### (a) Field sites

Data were collected from a ‘fished’ squaretail grouper spawning aggregation site (Bitra) during the new moon phase in January and from an ‘unfished’ site (Site 2) in February 2023 and 2024, in the Lakshadweep archipelago (details in electronic supplementary material 1, site characteristics). Historical data were also used from the fished site from January 2013 and 2014 (i.e. when it was in an unfished state [20]). Sampling, site and aggregation characteristics are summarized in table 1. Two prominent modes of active in-water reef fishing were observed at the fished site: hook-and-line and spearfishing, both involving fishers freediving in the water (R.K., personal observation, details in electronic supplementary material 1). Anti-predator behavioural responses have been demonstrated in response to both fishing scenarios [16,17,21].

**Table 1.** Site-level characteristics at the fished and unfished sites.

	Bitra 2013–2014 (unfished)	Bitra 2013–2014 (fished)	Site 2 2013–2014 (unfished)
sampling period	8–11 February 2013	19–23 January 2023	18–21 February 2023
	29 January–1 February 2014	9–13 January 2024	7–10 February 2024
peak aggregation day (new moon day)	10 February 2013	20 January 2023	20 February 2023
	29 January 2014	10 January 2023	8 February 2024
total aggregation area (m^2^)	16 000	16 000	30 000
core aggregation area (m^2^)	6000	6000	14 000
sampled area (m^2^)	2500	2500	3500
site depth range (m)	10–18	10–18	14–30
number of transects (50 × 10 m)	5	5	7
grouper density pertransect (on new moon day) (mean ± s.e., individuals.500 m^–2^)	162 ± 35.3	34.9 ± 15.1	128 ± 15.8
female : male sex ratio (on new moon day) (mean ± s.e.)	1.02 ± 0.43	0.23 ± 0.10	0.55 ± 0.04
structural complexity (vertical height in metres) (mean ± s.e.)	NA	50.85 ± 9.33	57.22 ± 9.08
live coral cover (percentage cover 1 m^−2^) (mean ± s.e.)	32 ± 3.21	27.33 ± 4.33	23.5 ± 4

NA - data not available

### (b) Squaretail grouper spawning aggregations

Squaretail groupers aggregate at both study sites for 5–6 days around the new moon from December to March [20]. Male groupers arrive 2–3 days earlier, establishing temporary territories (5–15 m^2^) that are vigorously defended from neighbours. Females typically join a day before the new moon and stay for 2–3 days after. The mating system is described as a ‘lek’ [20]. Given the males’ stationary behaviour and distinct body markings facilitating easy identification, our study focussed on analysing male reproductive behaviours.

### (c) Population surveys

The total aggregation area, including the ‘core’ lekking zone, was marked during snorkel surveys (refer to [20] and electronic supplementary material 2, methods). Groupers were surveyed within this core area by two observers (R.K., I..K.) using underwater visual sampling on SCUBA along permanent 50 × 10 m belt transects. The number of transects varied based on the core area size (*n* = 5 fished site, *n* = 7 unfished site). Abundance estimates per transect were used to calculate average site-level population density (grouper 500 m^−2^). Observers also recorded fish lengths (5-cm bins) and sex *in situ* [20]. Habitat characteristics such as structural complexity and percentage of live coral cover were assessed along three 50-m transects at the sites (details in electronic supplementary material 2).

### (d) Behavioural observations

Males were ‘paired’ with a female, if females were observed within male territories during focal follows and if displaying ‘mate-guarding’ behaviours, such as maintaining close contact with females or hovering nearby (electronic supplementary material 2).

#### (i) Reactive response

Reactive responses were measured at the fished and unfished sites during surveys done in 2023–2024. To determine the reactive fear response in territorial male groupers, we used ‘FID’, i.e. the distance at which the focal animal moves to avoid an approaching human observer [22]. FID is commonly used as an indicator to test for fish wariness in response to fishing [16]. We simulated predation risk by horizontally approaching, opportunistically selected territorial males (*n* = 72) on SCUBA at a steady pace from a standardized distance of 5 m [23]. The approaching observer dropped a marker when the male began to move away and the linear distance between the grouper position (prior to movement), and the marker was measured by a second observer with a tape measure. Time away from territory is known to positively influence territory intrusion in spawning territorial damselfish [24]. Therefore, we retreated 5 m from the male territory, immediately after the simulated disturbance to record how quickly an individual returns to its territory. In a pilot assessment of 20 males, over 70% of males returned within a minute, suggesting that immediate responses were critical for territory holders. We therefore used 120 s as a cut-off time in our extended dataset.

#### (ii) Proactive response

Focal video sampling was used to estimate time–activity budgets, comparing behaviour among the fished and unfished sites in 2023–2024, and within the fished site in 2013–2014 and 2023–2024. Observations were conducted on 108 territorial males over 3 days at each site, encompassing a day before, during, and after the new moon. Male behaviours were categorized into three states: ‘courtship’ (male–female interactions), ‘territorial aggression’ (male–male interactions) and ‘vigilance’ (electronic supplementary material 2, table S1). The proportion of time spent in each activity was calculated by dividing the total time in different states (seconds) by the total video length (seconds).

To determine whether fishing rather than site-level population and environmental factors drove time–activity budgets, we collected information on focal male body size (cm) and the number of males and females in an area of 25 m^2^ (i.e. competitor and mate density) from the centre of focal male territories. Habitat condition (live coral cover) and habitat structure (structural complexity) were ranked on a scale of 0–5 for each territory (electronic supplementary material 2, table S2), owing to their influence on anti-predator behaviours like shelter-seeking [25,26] and predator avoidance [27]. Context data were not available for the historical dataset (2013–2014), which was excluded from the analysis of drivers.

### (e) Statistical analysis

#### (i) Reactive response

We used bootstrap resampling (*R* = 1000) to generate 95% confidence intervals to compare mean FID and territory return times between paired and unpaired males at fished and unfished sites. Non-overlapping confidence intervals indicated significant differences [28]. Proactive response: we also employed bootstrap resampling to compare activity budgets between paired and unpaired males at fished and unfished sites and within the fished site (2013–2014 pre-fishing and 2023–2024 fishing scenario). Ternary plots illustrated trade-offs between courtship, aggression and vigilance.

#### (ii) Drivers of activity budgets

Bayesian zero-and-one inflated Dirichlet (zoid) regressions [29] were used to model the influence of fishing status, habitat condition, male size and social context on time–activity budgets. The analysis involved 70 individuals surveyed in 2023–2024. All analysis was conducted in R (electronic supplementary material 3, table S4).

## 3. Results

### (a) Behavioural responses among fished and unfished sites

The two aggregation sites varied in population characteristics: the unfished site had two times the size of the core aggregation area and 1.5 times the grouper density on new moon days as compared with the fished site. Female-to-male sex ratios were two times higher in the unfished site compared with the fished site (roughly one female per two males, electronic supplementary material 2, figures S1 and S2). The sites were comparable in terms of environmental characteristics like live coral cover and structural complexity (table 1).

We found no difference in FIDs among males that were paired with a female at the fished (mean 2.51 m ± 0.34 CI) and unfished sites (mean 2.50 m ± 0.28 CI), however, unpaired males fled much earlier upon approach at the fished (mean 3.26 m ± 0.30 CI) compared with the unfished site (mean 2.64 m ± CI 0.33, figure 1*a*). Although they fled at a comparable distance; paired males in the fished site took nearly 2.5 times longer (mean 32.08 s ± 9.87 CI) to return to their territories as compared with males at the unfished site (mean 13.48 s ± 2.71 CI, figure 2*b* and electronic supplementary material 3, table S1). Less than half of the unpaired males (42.42%, 14/33) returned to their original territories after the disturbance at the fished site, while two-thirds of the unpaired males (66.66%, 18/27) returned to their territories at the unfished site.

**Figure 3.**
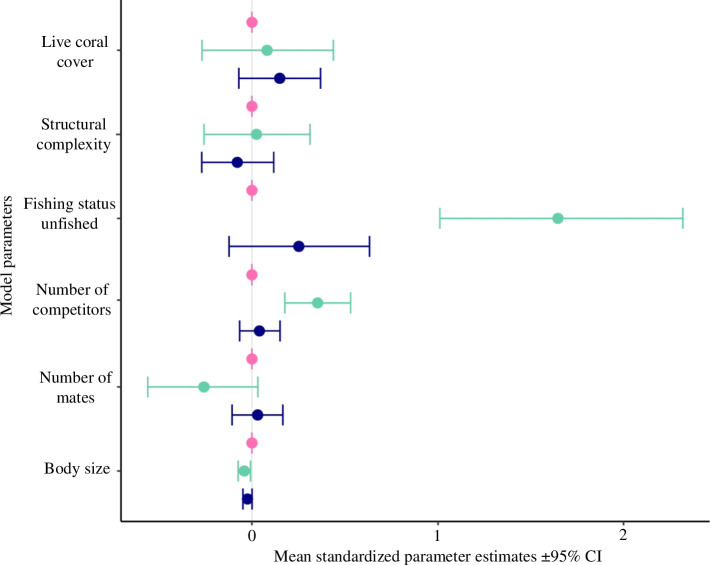
Zoid model parameters show how environmental (live coral cover, structural complexity, fishing exposure), social (number of mates and competitors) and individual (body size) factors affect male time allocation to courtship (pink), aggression (green) and vigilance (purple) at fished and unfished sites (*n* = 70).

Males spent nearly 10 times as long engaged in aggression at the unfished site (31.4%) compared with the fished site (4.68%), while the amount of time in courtship and vigilance did not vary between sites (figure 2*a*, electronic supplementary material 3, table S2). Males made different behavioural trade-offs at the fished and unfished sites. At the fished site, the distribution of most points along the axes of the ternary activity plot (figure 2*b*) rather than its centre, suggests that males made trade-offs between two behaviours at a time, rather than all three. Specifically, the primary trade-off was between courtship and vigilance, with little investment in aggression. In fact, 93% of males (39/43 males) exclusively dedicated their time to either courtship and/or vigilance, with 0% time engaged in aggressive interactions at the fished site. Contrastingly, at the unfished site, most males (17/31) exhibited a simultaneous trade-off among all three behaviours. Fishing status was the strongest predictor of differences in aggression between sites, followed by the number of mates, despite differences in environmental and social conditions around focal male territories (figure 3 and electronic supplementary material 3, table S3).

### (b) Behavioural responses within the fished site

The fished site saw a significant decline in population density and the female-to-male sex ratio over time. In a pre-fished condition, it had similar population characteristics to the unfished site (table 1, electronic supplementary material 2, figures S2 and S3).

Fishing exposure significantly altered time–activity budgets at the fished site; time in vigilance increased with fishing exposure, while aggression levels dropped significantly (figure 2). In a pre-fished condition, half of the males (19/33) exhibited all three behaviours simultaneously but after fishing began males showed trade-offs between vigilance and courtship only (figure 2), with very few investing in aggression. Time–activity budgets and behavioural trade-offs in the pre-fished condition were comparable to the unfished site (figure 2).

## 4. Discussion

Through observations and experimental simulations of predation risk, we demonstrate that anti-predator responses of male squaretail groupers vary based on courtship status and are heightened in spawning aggregations subjected to intense fishing. Unpaired males exhibit risk-averse behaviours, compromising reproductive opportunities by escaping early and avoiding returning to their mating territories at fished sites. In contrast, paired males display risk-prone behaviours even in fished areas. Further, increased fishing pressure makes fish more vigilant, resulting in trade-offs in territorial defence that can potentially impact mate selection cues. Altered behavioural priorities and anti-predator responses, triggered by fishing can mitigate short-term capture risk in aggregating fish but pose long-term threats to individual and population survival owing to risky decisions made during courtship.

Paired males displayed similar reactive anti-predator responses in both fished and unfished sites. According to life-history theory, an increased tendency for risk-taking is linked to higher individual reproductive efforts and the greater relative value of territories [30]. Our previous research showed that squaretail groupers with territories on the reef slope encountered more mating opportunities owing to female habitat preferences, indicating the high reproductive value of this habitat. However, males on the slopes also spent 25% of their time defending those territories, showing higher reproductive investment by individuals [20]. Higher reproductive value and investment are plausible explanations for why paired males adopted risk-prone strategies [31–33], as our study replicated sampling along the reef slope (12–20 m). Additionally, we found that all paired males returned to their original territories after the disturbance at both sites, but at the fished site, they returned with a significant delay. Although the success of long-term resident males in regaining their territory can be high against short-term intruders [34], this can rapidly decrease with increasing residence time of the intruder [24]. While we could not track the fate of all returning males, several males had to engage immediately in territorial defence with intruders after returning to their territories at both sites, underscoring the costs of being away from territories. Therefore, squaretail grouper males with delayed return times potentially run the risk of losing their territories and immediate mating opportunities associated with it in the fished site.

Paired males displayed risk-prone behaviours, whereas unpaired males in fished areas demonstrated risk aversion, either escaping early, returning late or not returning at all. Notably, aggregation densities at the fished site have plummeted by 70% since 2013, with fisheries now targeting groupers year-round, even outside aggregation periods and sites. Considering that squaretail groupers are long-lived species living up to 12 years [35], risk aversion may stem from learned responses to increased fishing exposure. Although FIDs were not tracked previously, we encountered difficulty replicating sampling protocols from the previous study (i.e. maintaining an observation distance at 4 m [20]), because most individuals were very flighty as reflected in the current FIDs averaging 3.25 m (3.12, 3.42). In fact, recent studies have shown longer FIDs in fish outside marine protected areas where fishing is chronic [16,17], consistent with our findings.

Time–activity budgets exhibited notable variations both within and among the aggregation sites. Intriguingly, the allocation of time to courtship behaviour remained consistent both within and across sites, indicating an optimization of reproductive efforts among individuals during the spawning aggregation. At the outset, vigilance levels showed no significant difference between sites, contrary to our expectations. However, prolonged monitoring at the fished site revealed increased vigilance behaviour, likely indicating heightened anti-predatory responses owing to increasing interactions with freediving fishers. At the unfished site, higher baseline vigilance may be owing to greater natural predator densities. A significant reduction in aggression was observed both within and between fished sites. Notably, contact aggression, such as jaw locking and biting, was absent, replaced entirely by non-contact aggression like size and colour displays at the fished site (electronic supplementary material 2, figure S3). This decreased intensity of aggression aligns with predictions of the economic defendability theory, postulating a decrease in territorial aggression in low-density conditions [36]. In our models, among social and environmental factors, fishing status was the strongest predictor of aggression levels, followed by the number of competitors in the immediate vicinity of the focal males. Reduced aggression is expected in environments classified as ‘high risk’, where increased energy expenditure and the risk of severe injury render aggressive males more vulnerable to predation, despite their potential for higher reproductive success [37]. Various other factors, unaccounted in this study, can influence aggression in territorial males. For instance, fishing may directly influence female mate choice and reproductive behaviours, which we did not examine. Evidence suggests that under high predation risk, females may become less selective [38], potentially reducing territorial aggression in males. Alternatively, the observed patterns may indicate a ‘timidity syndrome’, where individuals with bolder personalities, often associated with aggression, are more susceptible to fishing pressure and are thus removed from the population [39]. Decreased aggression can hold significant evolutionary implications [40] in mating aggregations; specifically a relaxation in sexual selection, which could have implications for mate choice, population fitness and long-term resilience [41]. Long-term differences in FID and activity budgets at the fished site highlight fishing’s significant impact on fish spawning aggregations. However, to generalize these behavioural variations and separate density-dependent effects from fear of fishing, further investigations are needed, such as monitoring behaviours by setting up temporal fishing closures at the fished site or conducting fishing simulations at the unfished site.

Our study reveals a paradox in aggregating fish behaviour, wherein individuals adopt risk-prone or risk-averse strategies based on their courtship status, each carrying distinct fitness costs. Behavioural trade-offs employed during reproductive phases are aimed at maximizing reproductive success and our findings of altered trade-offs behavioural trade-offs emphasize the need to consider the ecology of fear in a reproductive context.

## Data Availability

Datasets and R scripts used to generate figures and models are available from the Dryad Digital Repository [42]. Supplementary material is available online [43].
